# Transcutaneous spinal stimulation paired with visual feedback facilitates retention of improved weight transfer toward the affected side in people post-stroke

**DOI:** 10.1186/s12984-025-01673-1

**Published:** 2025-09-02

**Authors:** Hyunje Park, Beom-Chan Lee, Dimitry Sayenko, Sheng Li, Seoung Hoon Park

**Affiliations:** 1 Department of Health and Human Performance, University of Houston, Houston, TX 77204 USA; 2 Center for Neuromotor and Biomechanics Research (CNBR), University of Houston, Houston, TX 77204 USA; 3 Department of Neurosurgery, Center for Neuroregeneration, Houston Methodist Research Institute, Houston, TX 77030 USA; 4 Department of Physical Medicine and Rehabilitation, McGovern Medical School, University of Texas Health Science Center at Houston, Houston, TX 77030 USA; 5 The NeuroRecovery Research Center, TIRR Memorial Hermann Hospital, Houston, TX 77030 USA; 6 Department of Communication Sciences and Disorders, University of Houston, Houston, TX 77204 USA; 7 3875 Holman St. Rm 104 Garrison, Houston, TX 77204-6015 USA

**Keywords:** Stroke, Locomotion, Phasic transcutaneous spinal stimulation, Visual feedback, Weight transfer

## Abstract

**Background:**

Many individuals with hemiparesis after stroke are reluctant to transfer their body weight toward the affected side and rely more heavily on the unaffected leg while walking. Although visual feedback (VF) has been utilized to improve their weight transfer during walking, the effects of transcutaneous spinal stimulation (TSS) paired with VF during locomotor training in people post-stroke remain largely unexplored. The purpose of this study, therefore, was to determine whether phasic TSS paired with VF regarding weight shifting to the affected leg during treadmill walking would enhance weight transfer toward the affected side.

**Methods:**

Eleven individuals post-stroke participated in two testing conditions (i.e., TSS + VF & Sham + VF) in a random order with a 10-minute break. Each condition consisted of (1) walking 30 strides (baseline), (2) walking 100 strides with either TSS + VF or Sham + VF (adaptation), and (3) walking 30 strides (post-adaptation).

**Results:**

Participants exhibited similar changes in weight transfer toward the affected side during the early post-adaptation period for both testing conditions (*P* = 0.20). However, enhanced weight transfer was shown during the late post-adaptation period in the TSS + VF condition, compared with the Sham + VF condition (*P* = 0.019). Further, the TSS + VF condition induced greater enhancement in muscle activation of affected hip abductors (*P* = 0.002) during the early post-adaptation period, along with greater improvements in stance time of the affected leg (*P* = 0.04) and symmetry of stance time (*P* = 0.01), compared with the effect of the Sham + VF condition.

**Conclusion:**

Targeted spinal stimulation paired with visually guided weight transfer during locomotor training can facilitate improvements in weight transfer and enhanced use of the affected leg, which may lead to symmetrical gait patterns in individuals with hemiparesis after a stroke.

## Background

Many individuals who had a stroke encounter challenges in bearing weight on the affected leg due to muscle weakness, impaired motor control, and/or sensory deficits, leading to an increased reliance on the unaffected leg. This over-reliance on the unaffected leg during ambulation can lead to fatigue, ultimately resulting in reduced walking endurance and speed [1, 2]. In clinical settings, locomotor training utilizing a treadmill has been a prevalent method for improving walking capabilities in people post-stroke [3]. However, this traditional approach has not yielded the anticipated improvements in motor function of the affected leg [4], potentially due to the limited engagement of the affected leg stemming from compensatory strategies that favor the unaffected leg during ambulation [5, 6]. Therefore, it is crucial to devise an intervention strategy that promotes the use of the affected leg in stroke survivors while mitigating compensatory reliance on the unaffected side during treadmill training.

Application of transcutaneous electrical spinal stimulation (TSS) during treadmill training has the potential to augment the effectiveness of treadmill training by improving the control of the affected leg in individuals who experienced a stroke. TSS is a non-invasive technique that utilizes electrical currents to stimulate the spinal cord [7]. This method activates large-diameter afferent fibers [8–11], which can modulate the spinal cord excitability via interneuronal circuits and enhance the capacity for sensory information to contribute to locomotor control [12]. Furthermore, TSS has been shown to modulate the excitability of corticospinal pathways [13]. Consequently, the integration of TSS with locomotor training may lead to improved modulation of spinal circuitry and corticospinal pathways, ultimately facilitating motor control of the affected lower limb in post-stroke individuals.

The effects of spinal stimulation are significantly influenced by the specific location of the stimulation [7, 14, 15]. For example, research involving non-injured individuals has demonstrated that TSS applied between the T10 and L1 vertebrae, which approximately corresponds to the L2-L4 or L4-S2 spinal segments, results in a distinct sequence of neural activation within the proximal and distal motor pools [16]. Furthermore, TSS delivered between the spinous processes of the T12-L1 region, which aligns with the lumbosacral segments of the spinal cord, elicits a more pronounced response in the hip and leg muscles, thereby enhancing stability and forward progression, in comparison with TSS applied at the other spinal locations [16, 17]. Thus, in this study, we involve the application of TSS at the intervertebral space between T12 and L1 during treadmill walking in individuals post-stroke.

Phase-dependent spinal stimulation administered during motor bursting phases may enhance locomotor function in individuals post-stroke. This form of stimulation is characterized by its intermittent and rhythmically timed application, which aligns with the temporal dynamics of spinal cord neural circuits to target specific phases of movement [18]. This approach is consistent with Hebb’s Rule, which suggests that the synchronization of stimuli with particular neural activities strengthens neural connections, thereby facilitating neuroplasticity [19]. Research conducted on animal models has indicated that the implementation of phasic spinal stimulation during specific periods of motor bursting can influence the functions of the central pattern generator and spinal locomotion circuits on a cycle-by-cycle basis [18]. In this study, therefore, we administer TSS during the stance phase of the affected leg while participants ambulated on a treadmill.

In addition, application of visual feedback (VF) regarding weight transfer to the affected leg has the potential to encourage individuals with hemiparesis following a stroke to actively use the affected leg during walking [20]. VF has been utilized as a strategy to promote weight transfer to the affected leg within the context of stroke neurorehabilitation [21]. The incorporation of VF during treadmill training has been associated with improvements in gait speed, spatiotemporal gait parameters, and overall mobility [22, 23]. Moreover, the implementation of VF to modify gait-related outcomes may foster active engagement for persons who had a stroke [24], which is considered more effective than passive training in enhancing performance. Evidence suggests that VF can also encourage individuals with post-stroke hemiparesis to voluntarily shift body weight [21] and enhance the activation of hip joint muscles, such as hip abductors and adductors, which may contribute to improved mediolateral stability and increased weight bearing on the affected side [25]. Therefore, in this study, alongside the application of TSS, we provide VF about weight transfer to the affected leg during treadmill walking, with the aim of promoting voluntary weight shifting toward the affected leg and thereby enhancing its use, which may ultimately lead to improved locomotor performance.

Based on the existing literature, there is limited knowledge regarding the effects of TSS paired with VF on mediolateral weight transfer and the use of the affected leg during ambulation in individuals post-stroke. Although a few recent studies investigated the impact of non-invasive spinal stimulation combined with treadmill training on locomotion in people post-stroke, they did not specifically examine the use of the affected leg, focusing instead on gait symmetry and clinical outcomes [26, 27]. Furthermore, the effects of treadmill training combined with spinal stimulation did not exceed those of treadmill training alone [27], suggesting insufficient effects of spinal stimulation alone on locomotor training, and these studies utilized continuous timed application of stimulation [26, 27]. Therefore, the objective of this research is to evaluate the effects of phasic spinal stimulation combined with visually guided weight transfer toward the affected leg during treadmill walking in individuals post-stroke. We hypothesize that the application of phasic TSS, in conjunction with VF regarding weight transfer toward the affected leg during walking, would result in more significant improvements in mediolateral weight transfer toward the affected side, as well as increased activation of the muscles in the affected leg that facilitate mediolateral weight transfer, such as the hip abductors and adductors, compared with the effects observed under the sham TSS and VF condition.

## Methods

### Participants

Eleven individuals (61.73 ± 8.8 years, 6 females; see Table 1) with chronic stroke (> 6 months) were recruited for this study from the Center for Neuromotor and Biomechanics Research Database (> 150 subjects registered), the University of Texas Health Center (TIRR Memorial Hermann), and the local Houston community. The inclusion criteria for this study were as follows: (1) participants aged between 21 and 75 years; (2) a diagnosis of a single unilateral, supratentorial stroke of either ischemic or hemorrhagic etiology, confirmed through radiographic imaging; (3) the presence of weakness or paresis in the affected leg; and (4) the ability to stand and walk independently for a distance greater than 10 m, with the allowance for the use of assistive devices or ankle-foot orthoses (AFOs) if necessary. The use of an AFO was not considered an exclusion criterion. To ensure participant safety, individuals who routinely used an AFO were allowed to wear it during treadmill walking, as removing the device could increase the risk of unintended ground contact due to foot drop, thereby compromising safety [28]. Furthermore, the primary focus of this study was on the hip joint muscles, which play a key role in mediolateral weight transfer during gait. Given that the AFO primarily influences the activity of ankle dorsiflexors and plantarflexors [28], its use was not expected to significantly affect the outcome related to hip muscle function and kinematics [29, 30]. The age range of 21 to 75 years was selected to include adults capable of providing informed consent and engaging reliably in study procedures [31], while excluding individuals over 75 due to the increased prevalence of age-related conditions that may introduce confounding effects [32]. This range also aligns with age criteria commonly used in comparable studies [33–35]. The exclusion criteria included: (1) brainstem or cerebellar stroke; (2) a score of less than 24 on the Mini Mental State Examination [36]; (3) the presence of other neurological disorders, cardiorespiratory or metabolic conditions, or orthopedic issues that could impair ambulation; (4) uncontrolled hypertension, defined as systolic blood pressure exceeding 200 mmHg or diastolic blood pressure exceeding 110 mmHg; (5) administration of botulinum toxin within the six months prior to the study; (6) an inability to tolerate 30 min of treadmill walking, with sitting breaks as needed; and (7) complete loss of sensation in the affected foot. All participants verbally reported experiencing partial sensory loss in the paretic leg. The procedures were approved by the University of Houston Institutional Review Board and informed consent was obtained from all participants prior to data collection.

**Table 1:** Demographic information for the participants

*P*	Sex	Age (y)	Weight (kg)	Height (cm)	Post-injury (y)	Affected side	Assistive device	Self-selected comfortable speed (m/s)	Stimulation intensity (mA)	Session order
1	F	70	88.7	177	17	R	AFO	0.1	11.5	TSS + VF → Sham + VF
2	M	62	71.9	179	7	L	None	0.3	27.5	Sham + VF → TSS + VF
3	F	60	63.5	165.5	9	L	None	0.1	16.5	Sham + VF → TSS + VF
4	M	48	83.1	168	4	R	None	0.35	11	TSS + VF → Sham + VF
5	M	73	83	182	5	L	None	0.5	13.5	TSS + VF → Sham + VF
6	M	45	94.5	172	3	L	AFO	0.22	25	TSS + VF → Sham + VF
7	M	68	104	185	3	L	None	0.5	14	Sham + VF → TSS + VF
8	F	62	65.1	165	4	R	None	0.4	12.5	Sham + VF → TSS + VF
9	F	63	50.6	157	1	L	None	0.1	16.5	TSS + VF → Sham + VF
10	F	69	79.8	168	6	R	None	0.3	17.5	TSS + VF → Sham + VF
11	F	59	83.4	171	9	R	AFO	0.3	17.5	Sham + VF → TSS + VF

Demographic information for the participants. Abbreviations: P, participants; y, years; M, male; F; female; L, left; R, right; AFO, ankle foot orthosis. TSS + VF, transcutaneous spinal stimulation and Visual feedback; Sham + VF, Sham stimulation and Visual feedback

### Apparatus

**Fig. 1:**
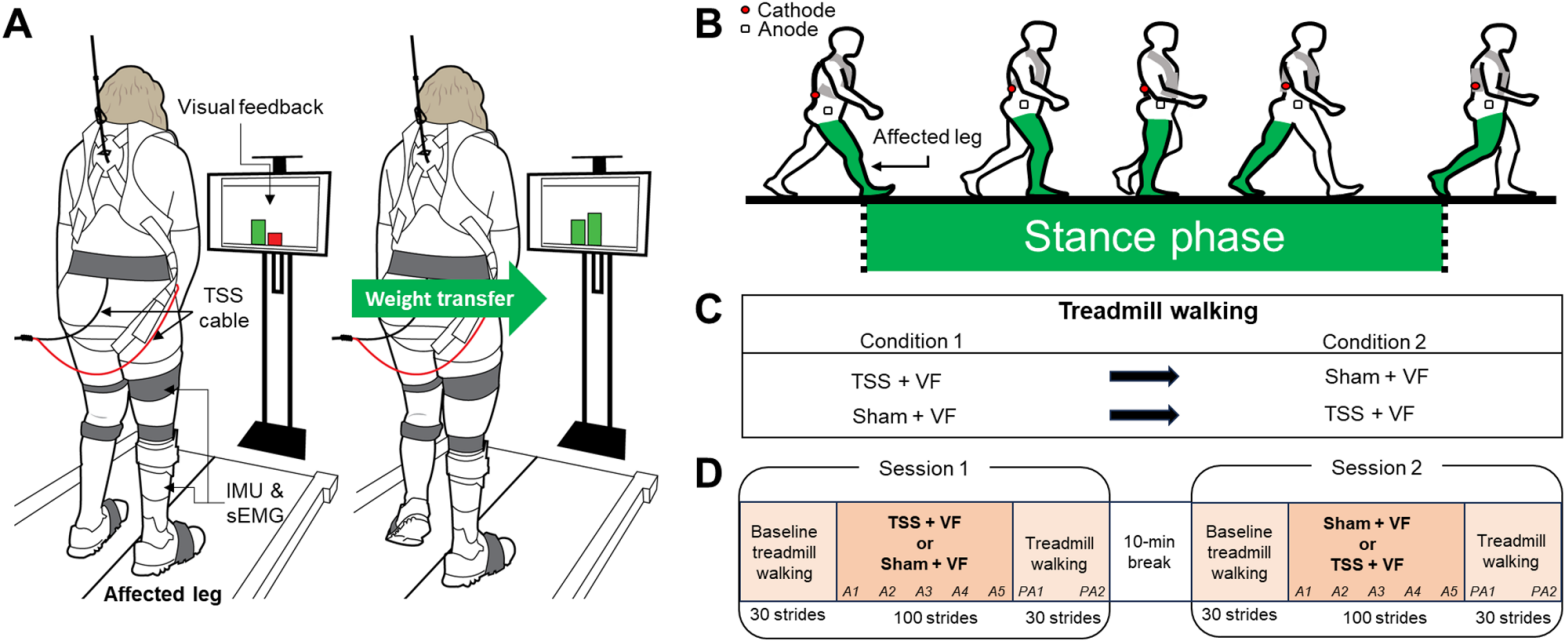
Experimental setup and protocol. **(A)** Participants walked on a dual-belt instrumented treadmill. During treadmill walking, transcutaneous spinal stimulation was applied via a cable-driven stimulator at predetermined stimulation intensity and concurrently visual feedback on weight transfer toward the affected leg was presented on a monitor. We recorded muscle activities from the affected leg using wireless sEMG sensors and positional signals with IMU sensors during treadmill walking. **(B)** In the TSS + VF condition, the transcutaneous spinal stimulation was applied to the designated location (cathode, T12-L1; anode, anterior iliac crest of the affected side) during the stance phase of the affected leg. **(C)** Participants completed two testing conditions (i.e., TSS + VF vs. Sham TSS + VF), which were randomly ordered (*n* = 6, TSS + VF to Sham + VF; *n* = 5, Sham + VF to TSS + VF). **(D)** During each condition, participants performed the treadmill walking task: 30 strides baseline treadmill walking; 100 strides treadmill walking with either “TSS + VF” or “Sham + VF” (adaptation period); 30 strides walking (post-adaptation period). Abbreviation: TSS, transcutaneous spinal stimulation; VF, visual feedback; sEMG, surface electromyography; IMU, inertial measurement unit; A1-5, adaptation periods; PA1, early post-adaptation period; PA2, late post-adaptation period

An instrumented dual-belt treadmill (Fig. 1A) was utilized in this study (Bertec Corporation, Columbus, OH, USA). This commercially available programmable treadmill system is equipped with two force plates located beneath each belt for precise measurement of ground reaction forces during walking. To provide TSS and VF during a specific gait phase (i.e., stance phase), we developed custom software using Microsoft Visual C++. Our software operated as a real-time, multi-threaded application that sampled vertical ground reaction force (vGRF) at a frequency of 100 Hz. Using the sampled vGRF data, our software detected gait events (i.e., heel strike and toe-off) based on a threshold of 5% of vGRF normalized to the participant’s body weight [37–39]. Subsequently, our software identified each gait cycle for precise application of TSS and VF. Particularly, our software triggered electrical stimulation during a stance phase of the affected leg, corresponding to a gait cycle between 10% and 60% (Fig. 1B). We used a biphasic constant current stimulator (DS8R; Digitimer, Welwyn Garden City, Herts, UK) to deliver TSS using two surface electrodes (area: 12.9 cm^2^). The cathode was placed along the midline of the spine at the intervertebral space between T12 and L1, and the anode was placed over the anterior iliac crest on the affected side.

VF was delivered via a 32-inch monitor (ViewFinity UJ59; Samsung Electronics America) positioned in front of the treadmill. Our software displayed the vGRF of the affected leg as a dynamic bar graph (Fig. 1A). Specifically, vGRF was displayed on a monitor as a dynamic bar chart, where one bar continuously updated and changed length based on the incoming vGRF of the affected leg in real time. The second bar, a fixed, green-colored target, was set based on the subject’s body weight. If a participant was unable to reach the target, the target value was gradually reduced in decrements of 0.5% or 1% (e.g., 99.5%, 99%, 98.5% of body weight, etc.) until a level was identified that the participant could reach with effort. This offered an immediate visual representation of mediolateral weight transfer performance of the affected leg. When the vGRF of the affected leg fell below the target, the bar graph appeared red, indicating insufficient weight shifting toward the affected side (Fig. 1A; left). Conversely, when the vGRF of the affected leg exceeded the target, the bar graph turned green (Fig. 1A; right), indicating improved weight transfer toward the affected side.

### Experimental protocol

All participants completed two testing conditions during a single day laboratory visit. The order of the two testing conditions was randomized among participants (6 participants: TSS + VF ◊ Sham + VF; 5 participants: Sham + VF ◊ TSS + VF; Fig. 1C). Participants, particularly those with lower walking function, might experience fatigue during testing. To mitigate this, we incorporated a 10-minute seated break between the two testing conditions as a washout period. This intermission duration was determined based on prior studies [35, 40–43]. In each condition, participants walked 160 strides on the dual-belt instrumented treadmill. The treadmill walking procedures for each condition included the following steps (Fig. 1D): (1) baseline walking for 30 strides; (2) walking with the application of either ‘TSS + VF’ or ‘Sham + VF’ for 100 strides, defined as the adaptation period; (3) walking without TSS and VF for 30 strides, defined as the post-adaptation period. Prior to the dual-belt treadmill walking test, participants were informed that TSS + VF would be applied during the adaptation period. We did not provide further specific instructions regarding their actions while walking on the treadmill, allowing for a natural response. Participants walked on the treadmill at their normal, comfortable gait speed in each condition, which was determined at the start of the initial testing session. The treadmill speed began at 0.05 m/s and was gradually increased in 0.05 m/s increments, with additional adjustments of 0.01 m/s made as needed. At each adjusted speed, participants walked approximately 10 strides to confirm whether the speed was comfortable and representative of their normal gait. Once a self-selected speed was confirmed, this speed was used consistently across all testing sessions. Additionally, participants completed a practice trial of approximately 10 strides prior to the first session to familiarize themselves with VF.

For the ‘TSS + VF’ condition (Fig. 1C), phase-dependent TSS was applied during the stance phase of the affected leg, using biphasic rectangular pulses (0.5 ms duration) at a frequency of 30 Hz, with intensities ranging from 11 to 27.5 mA. To determine the appropriate stimulus intensity, we administered TSS to the T12-L1 region and recorded evoked motor responses in the affected leg muscles—rectus femoris (RF), medial hamstring (MH), tibialis anterior (TA), soleus (SOL), and medial gastrocnemius (MG)—using EMG [44, 45]. The stimulation intensity was adjusted between 80 and 120% of the common motor threshold (i.e., TSS intensity that elicited responses in all recorded muscles), depending on individual tolerance. If participants could not tolerate this intensity, we adjusted it to a well-tolerated level. In addition to TSS, real-time VF on weight transfer toward the affected leg was provided, as illustrated in Fig. 1A.

For the Sham + VF condition, each participant followed the same protocol as described above, with the exception of the presence of sham TSS during the adaptation period (see Fig. 1D). In this condition, the electrodes were positioned identically, and the stimulation intensity was set to match that used during the TSS + VF condition. The intensity was gradually reduced to zero over the initial 10-second period [46, 47].

Prior to data collection, the participants walked on a treadmill at their maximal comfortable speed for 30 strides as a one-time trial. The electromyography (EMG) data collected during this session were used for the normalization of EMG signals in the two testing conditions. Thereafter, all treadmill walking during the two testing sessions was performed at each participant’s predetermined normal comfortable speed.

### Data collection

In each trial, muscle activity from TA, MG, SOL, MH, RF, vastus medialis (VM), hip adductor (ADD; adductor magnus), and hip abductor (ABD; gluteus medius) in the affected leg was measured using wireless surface EMG electrodes (Trigno, Delsys, Inc., Natick, MA, USA). EMG signals were recorded using the EMGworks Acquisition software (Delsys, Inc., Natick, MA, USA) at a sampling frequency of 1,926 Hz. In addition, vGRF data and gait events (i.e., heel strike and toe-off) were recorded by our custom software at a sampling frequency of 100 Hz.

### Data analysis

All EMG data, vGRF data, and gait events were analyzed using custom-written programs in MATLAB (MathWorks, MA, USA). The EMG data were resampled at 500 Hz, high-pass filtered at 10 Hz, and notch-filtered between 59 and 61 Hz and between 119 and 121 Hz to eliminate electrical noise. Subsequently, the processed EMG data were rectified and smoothed using a low-pass filter set at 20 Hz (fourth-order Butterworth). Following this processing, the EMG data were segmented into distinct gait cycles based on heel strike and toe-off event data, and normalized to the peak values of each muscle’s activity while participants walked at their maximum walking speed. Finally, the integrals of muscle activity during the stance phase of gait were calculated.

The vGRF signals were low-pass filtered at 10 Hz using a second-order Butterworth filter to attenuate high-frequency noise components. The filtered vGRF data were segmented into discrete gait cycles, defined from heel strike to the subsequent toe-off event. For each gait cycle, the peak vGRF value was extracted and normalized to the participant’s body weight, expressed as a percentage to account for inter-subject variability. Additionally, we counted the number of steps taken by the affected leg with enhanced weight transfer (toward the affected side) that exceeded the baseline level (i.e., at least one standard deviation above the baseline average of the vGRF for the affected leg) for each condition.

Stance time was defined as the duration during which a foot remains in contact with the ground, encompassing the interval from the initial ground contact to the final release [48]. The asymmetry index of stance time was quantified as follows [49]:

$$\:The\:asymmetry\:index\:=\:\left|\frac{affected-unaffected}{affected+unaffected}\:\right|\times\:100$$

Where affected is the stance time of the affected leg and unaffected is the stance time of the unaffected leg.

vGRF, EMG integrals, stance time, and stance time asymmetry during the following eight subintervals of the treadmill walking were calculated and compared across the two testing conditions or the different time points within each condition. The subintervals were: the 30 steps during baseline (B); the first 5 steps (A1), middle 5 steps (A3), and last 5 steps (A5) during the adaptation period; the 5 steps in the middle (A2) between A1 and A3, and the 5 steps in the middle (A4) between A3 and A5 during the adaptation period; the first 5 steps (PA1) and last 5 steps (PA2) during the post-adaptation period. To compare changes in vGRF, integrated EMG, stance time, and stance time asymmetry between the two testing conditions, the average of each variable during the baseline (i.e., B) was subtracted from that during the early (PA1) or late post-adaptation period (PA2).

### Statistical analysis

A two-way analysis of variance (ANOVA) with repeated measures was conducted to examine the interactions between condition (two conditions: TSS + VF and Sham + VF) and time (three time points: baseline, PA1, and PA2) regarding vGRF, muscle activity, stance time, and stance asymmetry during treadmill walking. Post-hoc tests with Bonferroni correction were performed only when the interaction effect was significant. We selected the time point PA1 (early post-adaptation) to investigate the immediate behavioral and neuromuscular responses to TSS + VF or Sham + VF, and the time point PA2 (late post-adaptation) to assess the retention of motor learning induced by each testing condition.

Paired t-tests were employed to compare changes in vGRF, muscle activity, stance time, and stance asymmetry from baseline to the early and late post-adaptation periods between the two conditions. Additionally, a linear regression model was utilized to assess the correlation (R^2^) between the number of steps demonstrating an enhanced weight shift toward the affected side and the change in weight transfer to the affected leg from baseline to the late post-adaptation period.

The IBM SPSS Statistics 22.0 statistical package (IBM Corp., Armonk, NY, USA) was utilized for the analysis. The alpha level for all statistical tests was set at 0.05. Data are presented as mean ± standard deviation in the text and as mean ± standard error of the mean in the figures.

## Results

### Vertical ground reaction force during the treadmill walking

**Fig. 2:**
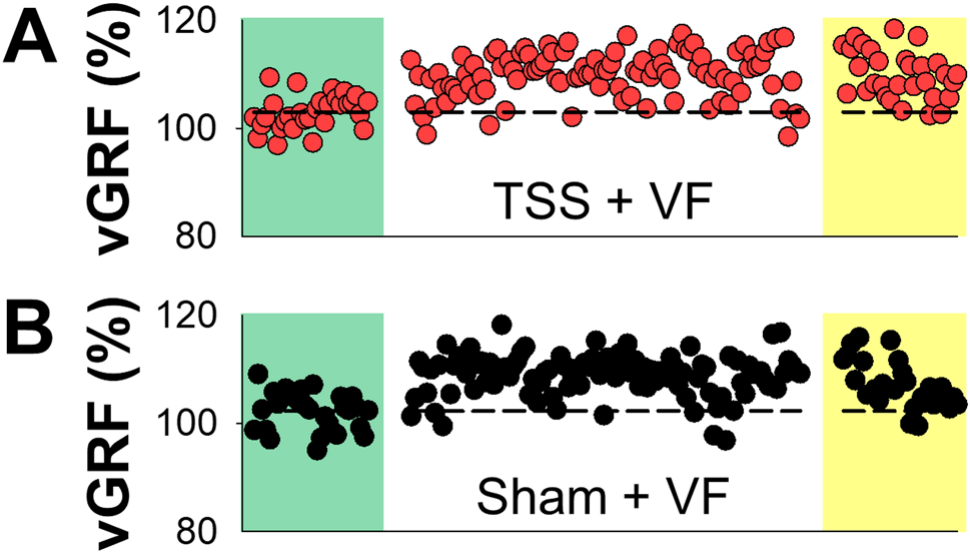
Stride-by-Stride vGRF of the affected leg from one representative participant for the TSS + VF (**A**) and Sham + VF (**B**) conditions. Abbreviation: vGRF, vertical ground reaction force; TSS, transcutaneous spinal stimulation; VF, visual feedback

vGRF toward the affected leg during treadmill walking for one typical participant under the TSS + VF and Sham + VF conditions is illustrated in Fig. 2. In the TSS + VF condition, the participant showed a noticeable increase in vGRF throughout the adaptation period. This elevated vGRF was also observed during the early post-adaptation period and was maintained to some extent until the conclusion of the post-adaptation period (Fig. 2A). In the Sham + VF condition, a similar increase in vGRF occurred during both the adaptation and early post-adaptation periods, akin to the TSS + VF condition (Fig. 2B). Then, the increased vGRF was returned to a level that was comparable to the baseline level during the mid-to-late post-adaptation period.

**Fig. 3:**
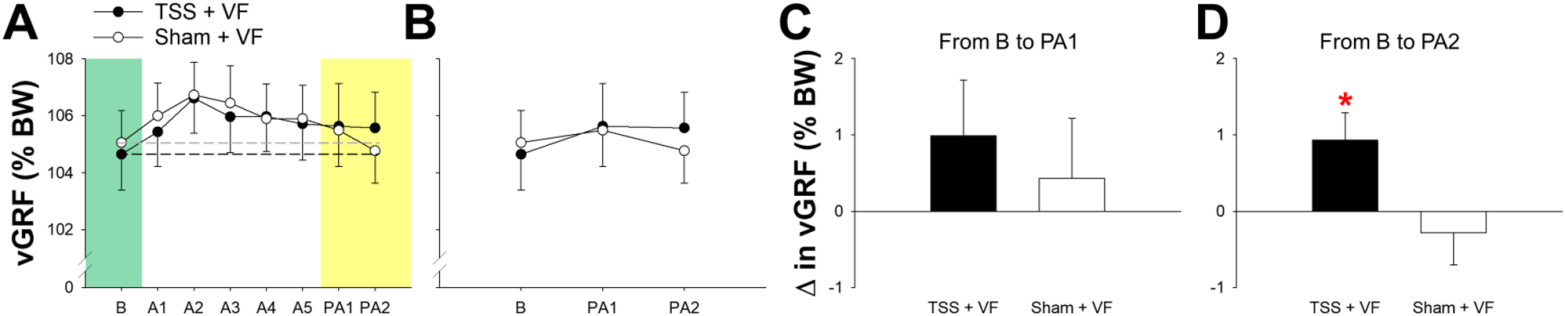
Group average of vGRF of the affected leg during treadmill walking. Data shown in column **A** were the average of vGRF during baseline (**B**), the adaptation periods (A1-A5), and the post-adaptation period (PA1-2). Horizontal dashed lines indicate the baseline average for each condition. Data shown in column **B** were the average of vGRF during baseline (**B**), and the post-adaptation period (PA1-2). The average of change in vGRF from baseline to the post-adaptation period (PA1-2) was shown in columns **C** and **D**. Abbreviation: vGRF, vertical ground reaction force; BW, body weight; B, baseline; A1-5, adaptation period; PA1, early post-adaptation period; PA2, late post-adaptation period; $\:\varDelta\:$, changes. Asterisks (*) indicate significant difference

The group average of vGRF from the affected leg during treadmill walking is presented in Fig. 3. A two-way ANOVA with repeated measures indicated that the condition (TSS + VF vs. Sham + VF) and time (baseline, PA1, and PA2) interaction was not significant for vGRF [F(2,20) = 3.042, *P* = 0.07].

Changes in vGRF from baseline to PA1 were not significantly different between the TSS + VF condition and the Sham + VF condition [t(10) = 1.324, *P* = 0.215]. However, changes in vGRF from baseline to PA2 were significantly greater for the TSS + VF condition than for the Sham + VF condition [t(10) = 2.807, *P* = 0.019].

**Fig. 4:**
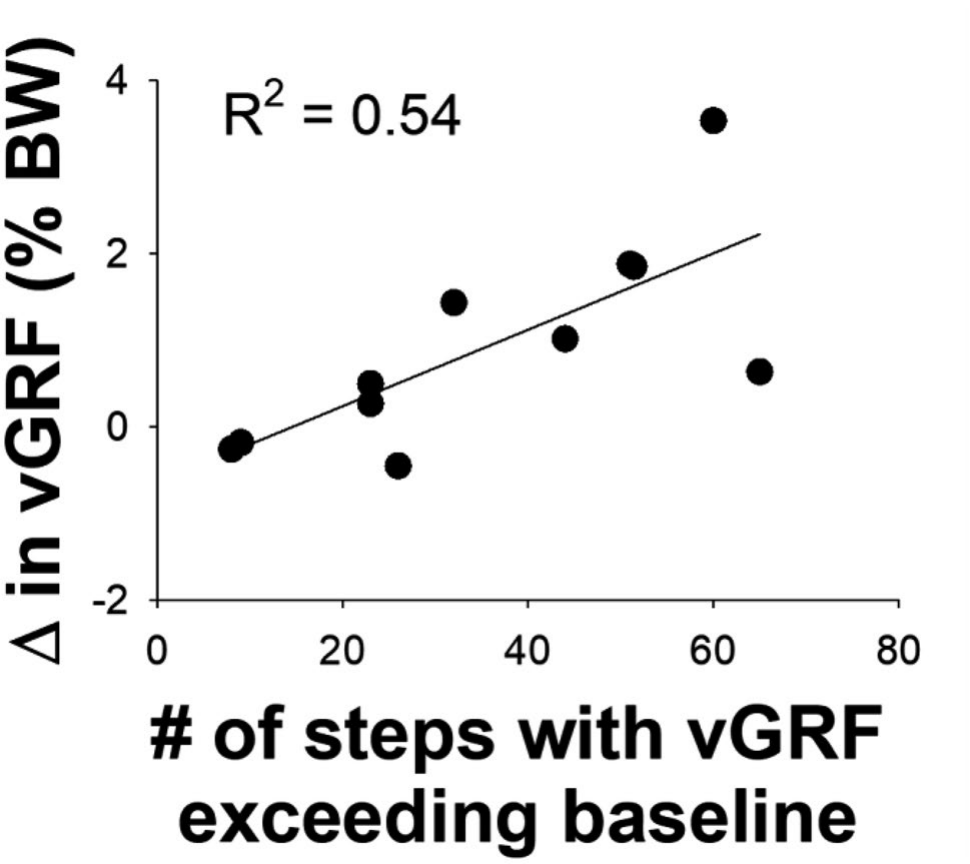
Correlation between the change in weight transfer toward the affected side from baseline to the late post-adaptation period and the number of the affected leg steps with enhanced weight transfer (toward the affected side) that exceeded the baseline level for the TSS + VF condition. Abbreviation: vGRF, vertical ground reaction force; #, number; BW, body weight

In the TSS + VF condition, participants who exhibited a greater number of steps with improved weight transfer toward the affected side showed greater improvements in weight transfer from baseline to the late post-adaptation period (R^2^ = 0.54, Durbin-Watson = 1.79, *P* = 0.01; Fig. 4).

### Muscle activity of the affected leg during the treadmill walking

**Fig. 5:**
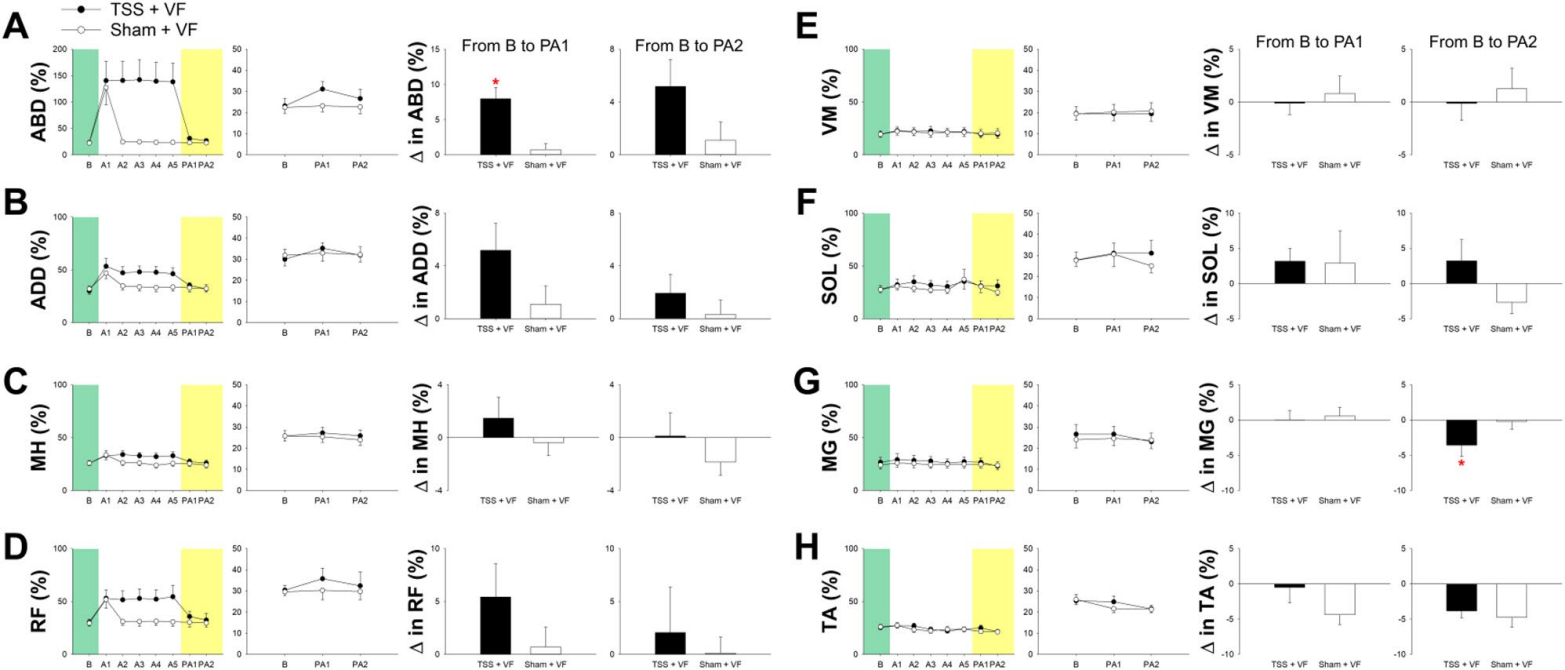
Group average of integrated EMG of the affected leg during treadmill walking. Data shown in the first column of each category were the average of integrated EMG during baseline (**B**), the adaptation period (**A1**-**A5**), and the post-adaptation period (PA1-2). Data shown in the second column of each category were the average of integrated EMG during baseline (**B**), and the post-adaptation period (PA1-2). Data shown in the third and fourth columns of each category were the average of change in integrated EMG from baseline to the post-adaptation period (PA1-2). Abbreviation: ABD, hip abductor; ADD, hip adductor; MH, medial hamstring; RF, rectus femoris; VM, vastus medialis; SOL, soleus; MG, medial gastrocnemius; TA, tibialis anterior; B, baseline; **A1-5**, adaptation period; PA1, early post-adaptation period; PA2, late post-adaptation period; ∆, changes. Asterisks (*) indicate significant difference

The group average of integrated EMG from the eight muscles in the affected leg is presented in Fig. 5. We analyzed EMG data from eleven participants across eight muscles (TA, MG, SOL, VM, RF, MH, ADD, and ABD). However, the data for the ABD muscle from one participant were heavily contaminated with extraneous electrical noise and were therefore excluded from the analysis. A two-way ANOVA with repeated measures revealed a significant interaction between condition (TSS + VF vs. Sham + VF) and time (baseline, PA1, and PA2) for the muscle activities of ABD [F(2,18) = 7.468, *P* = 0.004] and MG [F(2,20) = 4.035, *P* = 0.034]. Post hoc analysis using the Bonferroni correction (three comparisons conducted) indicated no significant differences in muscle activation for ABD (*P* ≥ 0.018) and MG (*P* ≥ 0.242) between the two testing conditions. The interaction between condition and time for the activities of other muscles was not significant [F(2,20) < 2.100, *P* ≥ 0.149].

Changes in ABD muscle activation from baseline to the early post-adaptation period were significantly greater for the TSS + VF condition than for the Sham + VF condition [t(9) = 4.181, *P* = 0.002]. In contrast, changes in ABD muscle activation from baseline to the late post-adaptation period did not show a significant difference between the two testing conditions [t(9) = 1.512, *P* = 0.165]. Changes in ADD muscle activation from baseline to the early post-adaptation period were not statistically significant for either testing condition [t(10) = 1.712, *P* = 0.118]. Changes in ADD muscle activation from baseline to the late post-adaptation period did not differ significantly between the two conditions [t(10) = 0.964, *P* = 0.358]. Changes in MG muscle activation from baseline to the early post-adaptation period were not significantly different between the two testing conditions [t(10) = -0.475, *P* = 0.645], but those from baseline to the late post-adaptation period were significantly different between the two testing conditions [t(10) = -2.671, *P* = 0.023]. Changes in MH, RF, VM, SOL, and TA muscle activation from baseline to the early or late post-adaptation period were not significantly different between the two conditions [*P* ≥ 0.065].

### Stance time and asymmetry during the treadmill walking

**Fig. 6:**
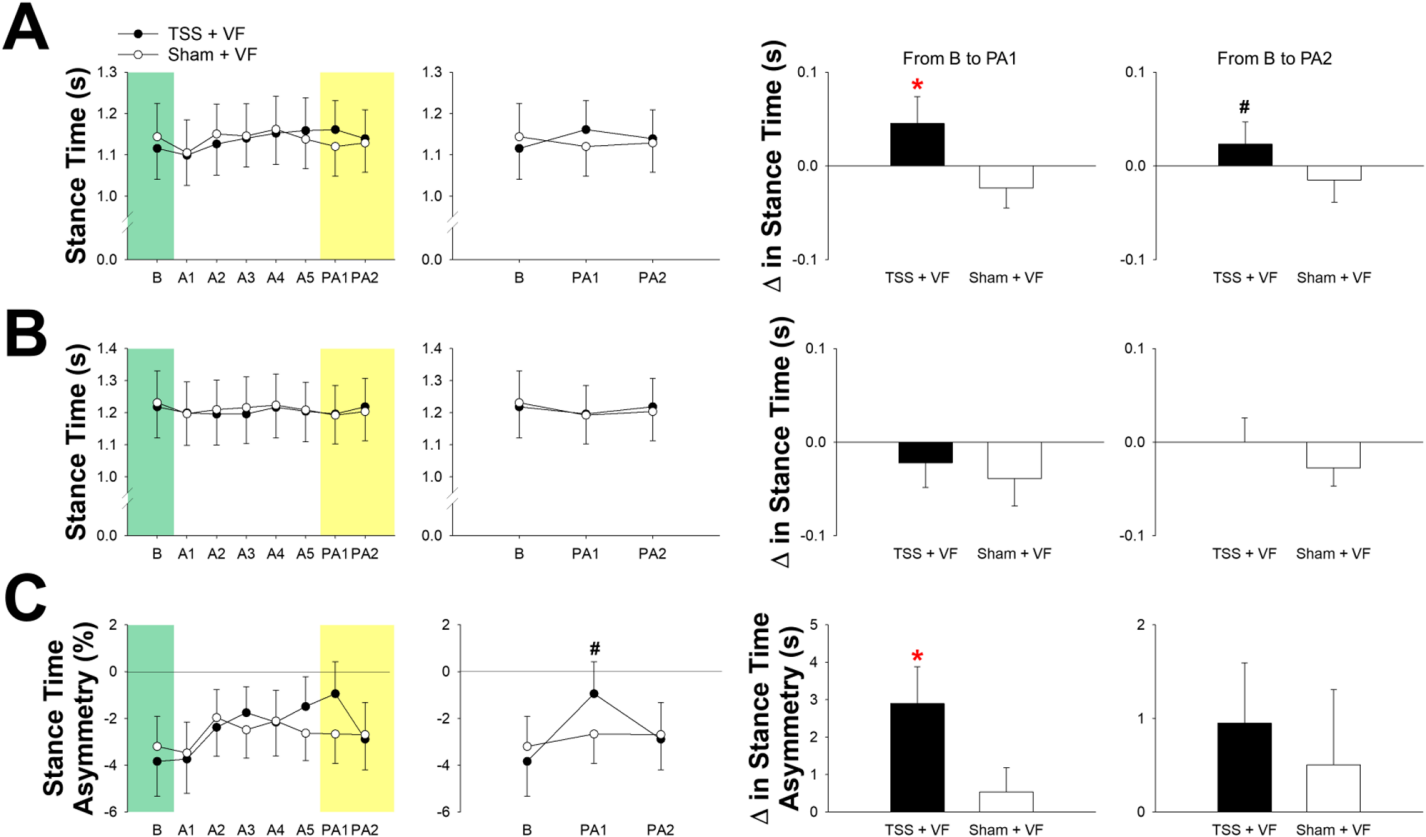
The stance time of the affected leg (**A**), that of the unaffected leg (**B**), and asymmetry of stance time (**C**) during treadmill walking. Data shown in the first column of each category were the average of stance time/stance time asymmetry during baseline (**B**), the adaptation period (**A1-A5**), and the post-adaptation period (PA1-2). Data shown in the second column of each category were the average of stance time/stance time asymmetry during baseline (**B**), and the post-adaptation period (PA1-2). Data shown in the third and fourth columns of each category were the average of change in stance time/stance time asymmetry from baseline to the post-adaptation period (PA1-2). Abbreviation: B, baseline; A1-5, adaptation period; PA1, early post-adaptation period; PA2, late post-adaptation period; ∆, changes. Asterisks (*) indicate significant difference. Hash (#) indicates a certain trend toward significance (*P* = 0.06)

The group averages of the stance time of the affected and unaffected leg and its asymmetry are shown in Fig. 6. A two-way ANOVA with repeated measures showed that the condition (TSS + VF vs. Sham + VF) x time (baseline, PA1, and PA2) interaction was significant for stance time of the affected leg (F(2,20) = 4.007, *P* = 0.034), Fig. 6A (2nd column). Post hoc analysis with the Bonferroni correction (three comparisons conducted) revealed no significant difference in stance time of the affected leg between the TSS + VF condition and the Sham + VF condition (*P* ≥ 0.18). Changes in stance time of the affected leg from baseline to early post-adaptation were significantly greater for the TSS + VF condition than for the Sham + VF condition [t(10) = 2.301, *P* = 0.044]. Changes in stance time from the baseline to the late post-adaptation period were not statistically different [t(10) = 2.066, *P* = 0.066].

A two-way ANOVA with repeated measures showed that the condition (TSS + VF vs. Sham + VF) x time (baseline, PA1, and PA2) interaction was not significant for stance time of the unaffected leg (F(2,20) = 0.538, *P* = 0.592), Fig. 6B (2nd column). Changes in stance time of the unaffected leg from baseline to early and late post-adaptation were not significantly different between the two testing conditions [*P* ≥ 0.271].

A two-way ANOVA with repeated measures showed that the condition (TSS + VF vs. Sham + VF) x time (baseline, PA1, and PA2) interaction was significant for stance time asymmetry (F(2,20) = 4.612, *P* = 0.023), Fig. 6C (2nd column). Post hoc analysis with the Bonferroni correction (three comparisons conducted) revealed no significant difference in stance time asymmetry between the two conditions (*P* ≥ 0.06). Changes in stance time asymmetry from baseline to the early post-adaptation [t(10) = 2.970, *P* = 0.014] were significantly different between the two testing conditions, Fig. 6C (3rd column). Changes in stance time asymmetry from baseline to the late post-adaptation were not statistically different [t(10) = 1.508, *P* = 0.162].

## Discussion

In this study, we found that the application of TSS paired with VF during walking resulted in a retention of increased weight transfer toward the affected side, compared with the effect of the Sham and VF condition. Additionally, participants exhibited enhanced activation of paretic hip abductors, along with an increased stance time of the affected leg and improved symmetry in stance time, in comparison with the control condition. These findings provide novel evidence that the application of phasic TSS paired with VF on weight transfer toward the affected leg during walking is effective in improving mediolateral weight transfer toward the affected side and enhancing the use of the affected leg, which may contribute to improvements in stance time of the affected leg in people post-stroke.

### Spinal stimulation + visual feedback promote motor learning

An important finding of this study is that the application of phasic TSS paired with visually guided weight transfer during treadmill walking resulted in the retention of increased weight transfer toward the affected side, despite both testing conditions yielding similar immediate aftereffects. Individuals experiencing hemiparesis following a stroke often exhibit a compromised ability to shift body weight toward the affected leg [50–52]. In this study, the provision of VF offered concurrent information regarding weight transfer, which can be classified as a form of knowledge of performance, potentially eliciting cognitive engagement from participants. Specifically, by delivering real-time VF regarding weight shifting onto the affected leg during ambulation, participants may have consciously adjusted their body weight further toward the affected side. This intervention likely augmented the loading on the affected leg, which might enhance the afferent input from the Golgi tendon organs in the ankle extensor muscles, as well as the cutaneous afferent input from the plantar surface of the foot [53–56]. This is consistent with findings from previous studies demonstrating that providing VF during treadmill walking induces improvements in weight transfer toward the affected side, gait speed, spatiotemporal gait characteristics, and overall mobility [21–23]. Although the provision of VF may promote voluntary weight shifting in people with hemiparesis after a stroke [21], a significant number of individuals continue to exhibit inadequate weight transfer towards the affected side, primarily attributable to the weakness of the affected lower limb [42]. Additional spinal stimulation may further activate afferent fibers, as indicated in previous studies [8 – [11, 57]. The engagement of these afferent pathways through spinal stimulation could potentially enhance the convergence of sensory inputs on spinal somatosensory networks [58, 59], thereby modulating spinal cord excitability to a degree that allows for more sensory information to contribute to balance and locomotor control [12, 60]. Moreover, spinal stimulation applied between the spinous processes of the T12-L1, which correspond to the lumbosacral segments of the spinal cord, may elicit a more pronounced response in the hip and leg muscles, thereby promoting stability. Thus, the enhanced activation of the hip abductors observed in the spinal stimulation and VF condition may increase the mechanical stiffness of the hip joint [61], improving stabilization of the affected hip joint for weight-bearing activities and facilitating more effective weight transfer to the affected side. The increased activation of the affected leg muscles sustained throughout the adaptation period may promote the use of the affected leg through a use-dependent motor learning mechanism [62]. This mechanism, which is engaged during the adaptation to VF in conjunction with spinal stimulation, could play a crucial role in the retention of the enhanced weight transfer during the late post-adaptation [63]. Notably, the enhancement in weight transfer toward the affected side during the late post-adaptation period was correlated with the number of steps taken with the affected leg, where the weight transfer exceeded baseline levels during the adaptation period. This indicates that those participants who demonstrated a greater number of steps with improved weight transfer toward the affected side during the adaptation period exhibited more significant improvements in weight transfer toward the affected side during the late post-adaptation period. Consequently, pelvic movements that facilitated improved weight shifting toward the affected side during the adaptation period may serve as a vital factor in the learning of enhanced weight transfer toward that side [64]. This finding aligns with prior research suggesting that the repetition of specific movements with an intervention can alter movement patterns or muscle activity, with such behavioral or neuromuscular changes potentially persisting even after the cessation of the intervention [65]. Furthermore, evidence indicates that the repetition of reaching movements that necessitate obstacle avoidance can result in curved movement trajectories, which may be retained following the removal of the obstacle [66].

In this study, we applied spinal stimulation during the stance phase of the affected lower limb while participants walked on a treadmill. The stance phases while walking correspond to motor bursting activity, and synchronizing stimulation with specific neural events may enhance the convergence of sensory and descending inputs on the spinal networks [56, 57, 60]. Furthermore, phasic stimulation may help maintain the transmission of proprioceptive information to the spinal cord. Studies on continuous spinal stimulation – a conventional approach – suggest that it may disrupt neural network dynamics and hinder the transmission of proprioceptive signals to the spinal cord [14]. Thus, employing phasic spinal stimulation to minimize such interference could enhance control over motor neuron activity and promote the facilitation of walking [14]. By integrating visually guided weight transfer with phasic spinal stimulation, it may be possible to foster neural connectivity and reduce disruptions in proprioceptive feedback. This approach supports sensorimotor learning [67] and could lead to meaningful improvements in weight transfer toward the affected leg in individuals recovering from a stroke.

Furthermore, alterations in mediolateral weight transfer during the adaptation period were found to be similar across both testing conditions (refer to Figs. 2A, amp and B and 3A), suggesting that the adaptation process was primarily facilitated by visual biofeedback. Immediately after the termination of VF, participants exhibited an aftereffect characterized by an increased weight transfer towards the affected side, as illustrated in PA1, which implies the potential formation of an internal model [68]. Notably, the aftereffect observed in the Sham + VF condition dissipated, while the aftereffect in the TSS + VF condition persisted until the conclusion of the post-adaptation period. A common characteristic of error-based motor learning is that the aftereffects produced by this learning mechanism tend to be transient. This observation leads to the hypothesis that visual feedback may exert a significant influence on error-based motor learning, thereby resulting in the short-lived aftereffect associated with the Sham + VF condition. Conversely, once the error-based aftereffect diminished, the effects stemming from the use-dependent motor learning mechanism began to manifest, which typically exhibits greater retention. This finding aligns with previous research indicating that error-based and use-dependent learning can concurrently influence motor behavior, with participants initially displaying a brief error-based aftereffect followed by a more enduring use-dependent motor learning effect in both healthy individuals [63] and those who have experienced a stroke [40].

### Spinal stimulation + visual feedback induce symmetrical gait patterns

In this study, we found that participants exhibited a greater improvement in the stance time of the affected leg and in the symmetry of stance time from baseline to the early post-adaptation period in the TSS + VF condition, compared with the sham + VF condition (Fig. 6C). During the baseline assessment, nine participants demonstrated shorter stance times on the affected leg, while two participants showed longer stance times on the affected leg (as indicated in Table 2). This observation led us to hypothesize that the changes in stance time of the affected lower limb induced by spinal stimulation and VF might differentially influence the symmetry of stance time. In the TSS + VF condition, regardless of whether participants had shorter or longer stance times on the affected leg at baseline, they exhibited an increased stance time on the affected leg and a decreased stance time on the unaffected leg immediately following the cessation of spinal stimulation and VF. One possible mechanism for this phenomenon is that enhanced weight transfer toward the affected side may result in increased load bearing on the affected leg, potentially stimulating greater muscle activity in the hip abductors, hip adductors, knee flexors, and knee extensors, thereby prolonging the stance time of the affected leg. This is consistent with prior research conducted on animal models, which suggests that load afferents from cutaneous receptors on the plantar surface of the foot [69] or from extensor muscles [70] may delay the activation of leg flexors associated with the leg swing. Since nine participants initially walked with a shorter stance time on the affected leg at baseline, the increased stance time on the affected leg and the decreased stance time on the unaffected leg immediately after the intervention (i.e., early post-adaptation period) appeared to improve the symmetry of stance time. Conversely, these changes in stance time for the affected and unaffected legs seemingly worsened the asymmetrical stance time for the two participants who walked with a longer stance time on the affected leg during the baseline assessment (Table 2).

**Table 2:** Stance time and asymmetry of stance time during the treadmill walking task

Condition	Leg	B	PA1	PA2	Δ from B to PA1	Δ from B to PA2
**Stance time (Unaffected > Affected; 9)**						
TSS + VF	Affected (s)	1.17 (0.08)	1.21 (0.08)	1.19 (0.07)	0.04 (0.03)	0.02 (0.03)
	Unaffected (s)	1.30 (0.09)	1.28 (0.08)	1.30 (0.09)	-0.02 (0.03)	0.00 (0.03)
	Asymmetry (%)	-5.17 (1.47)	-2.82 (0.65)	-4.26 (1.16)	2.34 (1.12)	0.90 (0.75)
Sham + VF	Affected (s)	1.20 (0.08)	1.17 (0.07)	1.18 (0.07)	-0.03 (0.03)	-0.02 (0.03)
	Unaffected (s)	1.31 (0.09)	1.27 (0.09)	1.29 (0.09)	-0.04 (0.04)	-0.03 (0.02)
	Asymmetry (%)	-4.41 (1.22)	-3.88 (1.13)	-3.92 (1.36)	0.53 (0.67)	0.49 (0.94)
**Stance time (Unaffected < Affected; 2)**						
TSS + VF	Affected (s)	0.89 (0.13)	0.96 (0.12)	0.91 (0.11)	0.07 (0.01)	0.02 (0.02)
	Unaffected (s)	0.86 (0.15)	0.83 (0.11)	0.85 (0.10)	-0.03 (0.04)	0.00 (0.05)
	Asymmetry (%)	2.14 (1.21)	7.53 (0.22)	3.30 (0.35)	5.38 (1.00)	1.16 (1.56)
Sham + VF	Affected (s)	0.90 (0.16)	0.89 (0.15)	0.89 (0.12)	-0.01 (0.01)	-0.01 (0.04)
	Unaffected (s)	0.86 (0.16)	0.83 (0.11)	0.84 (0.10)	-0.03 (0.06)	-0.02 (0.07)
	Asymmetry (%)	2.27 (0.81)	2.81 (1.85)	2.82 (1.10)	0.54 (2.66)	0.55 (1.90)

Abbreviations: B, baseline; PA1, early post-adaptation; PA2, late post-adaptation; Δ, change. Numbers in parentheses indicate standard errors

The results of this study hold significant implications for locomotor rehabilitation in individuals who had a stroke. In clinical practice, locomotor training is frequently employed to enhance walking capabilities in individuals with hemiparesis following a stroke. Nonetheless, it has been observed that people post-stroke often compensate by relying more heavily on their unaffected leg for mobility, which may hinder the recovery of motor function in the affected leg. Our findings indicate that the application of TSS in conjunction with real-time VF regarding weight transfer to the affected side during locomotor training could facilitate improvements in mediolateral weight transfer and promote the use of the affected leg, thereby improving temporal gait symmetry. Thus, TSS can be readily integrated as a straight forward, non-invasive, and cost-effective method into locomotor training regimens for individuals post-stroke. TSS represents a non-invasive modality that allows flexible electrode placement and individualized modulation of stimulation intensity to optimize both patient comfort and therapeutic efficacy. Given its methodological and clinical advantages, TSS demonstrates substantial potential for practical implementation and effective integration within standard physical therapy protocols [71]. In clinical settings, implementing VF techniques may not be practical, as they typically require an instrumented treadmill or pressure insoles to measure body weight in real time, along with a system to visually display weight transfer on a monitor. In the absence of VF techniques, alternative forms of biofeedback, such as verbal or tactile feedback, can be more feasible to promote weight transfer to the affected side during walking in routine clinical practice. Clinicians or physical therapists may observe how patients shift their body weight toward the weaker side during walking and provide verbal feedback to encourage appropriate weight transfer. Tactile cues may also be employed. For example, asking the patient to touch the therapist’s hand with their pelvis to guide movement toward the desired direction. This approach is supported by previous studies indicating that verbal and tactile cues can effectively deliver real-time performance feedback, promoting corrective responses during treadmill walking [72, 73]. The insights gained from this study may assist clinicians and researchers in developing targeted intervention strategies aimed at improving weight transfer to the affected side in individuals with hemiparesis following a stroke.

This study has several limitations. In this study, we focused exclusively on short-term changes in muscle activation and motor performance. Our findings indicate that the combined application of spinal stimulation and visual biofeedback resulted in significant immediate neuromuscular effects and the retention of behavioral changes. This may establish a foundation for longer and more repetitive sessions of TSS combined with visual feedback for gait rehabilitation. Also, each testing session for the respective conditions was approximately four to six minutes in duration, which might be insufficient to elicit more enduring effects, such as adapted locomotor patterns and fatigue. A ten-minute seated intermission was implemented between the two testing sessions, and the sequence of the sessions was randomized across participants. Therefore, it is unlikely that carryover effects would systematically influence our findings. Another limitation of this study is the absence of an objective clinical assessment, such as the lower-extremity motor domain of the Fugl-Meyer Assessment (FMA), to characterize participants’ motor impairments. While participants were screened for functional ability through self-report and on-site verification of walking and standing capacity, the inclusion of a standardized assessment such as the FMA would have provided a more precise evaluation of motor function. Future studies involving longer-term interventions will incorporate the FMA to more accurately assess baseline motor impairments and evaluate changes in motor function over time.

## Conclusion

This study is the first to identify the effects of applying phasic TSS paired with visual biofeedback on weight transfer to the affected leg in individuals post-stroke. The repeated application of phasic spinal stimulation paired with visually guided weight transfer during walking proved beneficial in enhancing activation of muscles responsible for weight bearing on the affected leg, as well as enhancing weight transfer on the affected side in people post-stroke. Knowledge derived from this study offers a novel perspective on the advancement of locomotor training approaches aimed at improving motor function of the affected leg and overall walking capabilities in humans post-stroke.

The rehabilitation protocol developed in this study, which integrates spinal stimulation with visual biofeedback, presents a promising avenue for personalized gait rehabilitation. This adaptive gait training intervention is designed to respond in real-time to limb-specific alterations in gait among individuals who have experienced a stroke. The targeted spinal stimulation and visual biofeedback mechanisms ensure that rehabilitation efforts are concentrated on mitigating deficits in weight-bearing on the affected limb. From a clinical standpoint, the protocol may serve as a scalable, adaptable, and patient-centered solution for gait rehabilitation. Its incorporation into clinical practice has the potential to enhance post-stroke gait rehabilitation by providing precise, targeted interventions aimed at improving weight transfer to the affected side during ambulation.

## Data Availability

The data from the current study are available from the corresponding author on reasonable request.

## References

[1] de Haart M, et al. Recovery of standing balance in postacute stroke patients: a rehabilitation cohort study. Arch Phys Med Rehabil. 2004;85(6):886–95. 15179641 10.1016/j.apmr.2003.05.012

[2] Michael KM, Allen JK, Macko RF. Reduced ambulatory activity after stroke: the role of balance, gait, and cardiovascular fitness. Arch Phys Med Rehabil. 2005;86(8):1552–6. 16084807 10.1016/j.apmr.2004.12.026

[3] Franceschini M, et al. Walking after stroke: what does treadmill training with body weight support add to overground gait training in patients early after stroke?? Stroke. 2009;40(9):3079–85. 19556526 10.1161/STROKEAHA.109.555540

[4] Mehrholz J, Thomas S, Elsner B. Treadmill training and body weight support for walking after stroke. Cochrane database of systematic reviews, 2017(8). 10.1002/14651858.CD002840.pub4 PMC6483714 28815562

[5] Raja B, Neptune RR, Kautz SA. Coordination of the non-paretic leg during hemiparetic gait: expected and novel compensatory patterns. Clin Biomech (Bristol Avon). 2012;27(10):1023–30. 10.1016/j.clinbiomech.2012.08.005 PMC3535278 22981679

[6] Roelker SA, et al. Paretic propulsion as a measure of walking performance and functional motor recovery post-stroke: A review. Gait Posture. 2019;68:6–14. 30408710 10.1016/j.gaitpost.2018.10.027 PMC6657344

[7] Gerasimenko Y, et al. Initiation and modulation of locomotor circuitry output with multisite transcutaneous electrical stimulation of the spinal cord in noninjured humans. J Neurophysiol. 2015;113(3):834–42. 25376784 10.1152/jn.00609.2014

[8] Capogrosso M, et al. A computational model for epidural electrical stimulation of spinal sensorimotor circuits. J Neurosci. 2013;33(49):19326–40. 24305828 10.1523/JNEUROSCI.1688-13.2013 PMC6618777

[9] Rattay F, Minassian K, Dimitrijevic MR. Epidural electrical stimulation of posterior structures of the human lumbosacral cord: 2. quantitative analysis by computer modeling. Spinal Cord. 2000;38(8):473–89. 10962608 10.1038/sj.sc.3101039

[10] Gerasimenko YP, et al. Spinal cord reflexes induced by epidural spinal cord stimulation in normal awake rats. J Neurosci Methods. 2006;157(2):253–63. 16764937 10.1016/j.jneumeth.2006.05.004

[11] Hofstoetter US, et al. Periodic modulation of repetitively elicited monosynaptic reflexes of the human lumbosacral spinal cord. J Neurophysiol. 2015;114(1):400–10. 25904708 10.1152/jn.00136.2015 PMC4507962

[12] Edgerton VR, et al. Training locomotor networks. Brain Res Rev. 2008;57(1):241–54. 18022244 10.1016/j.brainresrev.2007.09.002 PMC2288528

[13] Steele AG, et al. Characterization of spinal sensorimotor network using transcutaneous spinal stimulation during voluntary movement Preparation and performance. J Clin Med. 2021;10(24):5958. 34945253 10.3390/jcm10245958 PMC8709482

[14] Formento E, et al. Electrical spinal cord stimulation must preserve proprioception to enable locomotion in humans with spinal cord injury. Nat Neurosci. 2018;21(12):1728–41. 30382196 10.1038/s41593-018-0262-6 PMC6268129

[15] Gerasimenko Y, et al. Transcutaneous electrical spinal-cord stimulation in humans. Annals Phys Rehabilitation Med. 2015;58(4):225–31. 10.1016/j.rehab.2015.05.003 PMC5021439 26205686

[16] Sayenko DG, et al. Spinal segment-specific transcutaneous stimulation differentially shapes activation pattern among motor pools in humans. J Appl Physiol. 2015;118(11):1364–74. 25814642 10.1152/japplphysiol.01128.2014 PMC4451290

[17] Tran K, et al. Multi-site lumbar transcutaneous spinal cord stimulation: when less is more. Neurosci Lett. 2024;820:137579. 38096973 10.1016/j.neulet.2023.137579 PMC10872491

[18] Vogelstein RJ, et al. Phase-dependent effects of spinal cord stimulation on locomotor activity. IEEE Trans Neural Syst Rehabil Eng. 2006;14(3):257–65. 17009484 10.1109/TNSRE.2006.881586

[19] Brown TH, Kairiss EW, Keenan CL. Hebbian synapses: biophysical mechanisms and algorithms. Annu Rev Neurosci. 1990;13(1):475–511. 2183685 10.1146/annurev.ne.13.030190.002355

[20] Liu J, et al. Comparison of the immediate effects of audio, visual, or audiovisual gait biofeedback on propulsive force generation in able-bodied and post-stroke individuals. Appl Psychophysiol Biofeedback. 2020;45:211–20. 32347399 10.1007/s10484-020-09464-1 PMC7447533

[21] Van Vliet PM, Wulf G. Extrinsic feedback for motor learning after stroke: what is the evidence? Disabil Rehabil. 2006;28(13–14):831–40. 16777770 10.1080/09638280500534937

[22] Drużbicki M et al. The efficacy of gait training using a body weight support treadmill and visual biofeedback in patients with subacute stroke: A randomized controlled trial. BioMed research international, 2018. 2018. 10.1155/2018/3812602 PMC5907400 29850509

[23] Lewek MD, et al. Use of visual and proprioceptive feedback to improve gait speed and Spatiotemporal symmetry following chronic stroke: a case series. Phys Ther. 2012;92(5):748–56. 22228605 10.2522/ptj.20110206 PMC3345339

[24] van Gelder LM, et al. The use of biofeedback for gait retraining: A mapping review. Clin Biomech Elsevier Ltd. 2018;59:159–66. 10.1016/j.clinbiomech.2018.09.020 30253260

[25] Pak N-W, Lee J-H. Effects of visual feedback training and visual targets on muscle activation, balancing, and walking ability in adults after hemiplegic stroke: A preliminary, randomized, controlled study. Int J Rehabil Res. 2020;43(1):76–81. 31633580 10.1097/MRR.0000000000000376

[26] Moon Y, et al. Noninvasive spinal stimulation improves walking in chronic stroke survivors: a proof-of-concept case series. Biomed Eng Online. 2024;23(1):38. 38561821 10.1186/s12938-024-01231-1 PMC10986021

[27] Awosika OO, et al. Backward locomotor treadmill training combined with transcutaneous spinal direct current stimulation in stroke: a randomized pilot feasibility and safety study. Brain Commun. 2020;2(1):fcaa045. 32954299 10.1093/braincomms/fcaa045 PMC7425394

[28] Geboers JF, et al. Immediate and long-term effects of ankle-foot orthosis on muscle activity during walking: a randomized study of patients with unilateral foot drop. Arch Phys Med Rehabil. 2002;83(2):240–5. 11833029 10.1053/apmr.2002.27462

[29] Zissimopoulos A, Fatone S, Gard S. Effects of ankle–foot orthoses on mediolateral foot-placement ability during post-stroke gait. Prosthet Orthot Int. 2015;39(5):372–9. 24878846 10.1177/0309364614534294

[30] Tyson S, Sadeghi-Demneh E, Nester C. A systematic review and meta-analysis of the effect of an ankle-foot orthosis on gait biomechanics after stroke. Clin Rehabil. 2013;27(10):879–91. 23798747 10.1177/0269215513486497

[31] Arnett JJ. Emerging adulthood: A theory of development from the late teens through the twenties. Am Psychol. 2000;55(5):469. 10842426

[32] Harada CN, Love MCN, Triebel K. Normal cognitive aging. Clin Geriatr Med. 2013;29(4):737. 24094294 10.1016/j.cger.2013.07.002 PMC4015335

[33] Hsu CJ, et al. Applying a pelvic corrective force induces forced use of the Paretic leg and improves Paretic leg EMG activities of individuals post-stroke during treadmill walking. Clin Neurophysiol. 2017;128(10):1915–22. 28826022 10.1016/j.clinph.2017.07.409 PMC5593794

[34] Hsu CJ, et al. Forced use of the Paretic leg induced by a constraint force applied to the nonparetic leg in individuals poststroke during walking. Neurorehabil Neural Repair. 2017;31(12):1042–52. 29145773 10.1177/1545968317740972 PMC6083449

[35] Park SH, et al. Targeted pelvic constraint force induces enhanced use of the Paretic leg during walking in persons post-stroke. IEEE Transactions on Neural Systems and Rehabilitation Engineering; 2020. 10.1109/TNSRE.2020.3018397 PMC7652375 32816677

[36] Folstein MF, Robins LN, Helzer JE. The mini-mental state examination. Arch Gen Psychiatry. 1983;40(7):812–812. 6860082 10.1001/archpsyc.1983.01790060110016

[37] Yoo D, Seo K-H, Lee B-C. The effect of the most common gait perturbations on the compensatory limb’s ankle, knee, and hip moments during the first stepping response. Gait Posture. 2019;71:98–104. 31031225 10.1016/j.gaitpost.2019.04.013

[38] Neckel ND, et al. Abnormal joint torque patterns exhibited by chronic stroke subjects while walking with a prescribed physiological gait pattern. J Neuroeng Rehabil. 2008;5:1–13. 18761735 10.1186/1743-0003-5-19 PMC2553074

[39] Rouhani H, et al. Heel strike detection using split force-plate treadmill. Gait Posture. 2015;41(3):863–6. 25800003 10.1016/j.gaitpost.2015.02.021

[40] Park SH, et al. Enhanced phasic sensory afferents paired with controlled constraint force improve weight shift toward the Paretic side in individuals post-stroke. J Stroke Cerebrovasc Dis. 2023;32(4):107035. 36739709 10.1016/j.jstrokecerebrovasdis.2023.107035 PMC10065899

[41] Park SH et al. Increased motor variability facilitates motor learning in weight shift toward the Paretic side during walking in individuals post-stroke. Eur J Neurosci, 2021. 10.1111/ejn.15212 33783888

[42] Hsu CJ, et al. Use of pelvic corrective force with visual feedback improves Paretic leg muscle activities and gait performance after stroke. IEEE Trans Neural Syst Rehabil Eng. 2019;27(12):2353–60. 31675335 10.1109/TNSRE.2019.2950226 PMC6939618

[43] Wu M, Hsu CJ, Kim J. Forced use of Paretic leg induced by constraining the non-paretic leg leads to motor learning in individuals post-stroke. Exp Brain Res. 2019;237(10):2691–703. 31407027 10.1007/s00221-019-05624-w PMC6755123

[44] Roberts BW, et al. Transcutaneous spinal cord stimulation improves postural stability in individuals with multiple sclerosis. Multiple Scler Relat Disorders. 2021;52:103009. 10.1016/j.msard.2021.103009 34023772

[45] Manson GA, et al. Transcutaneous spinal stimulation alters cortical and subcortical activation patterns during mimicked-standing: A proof-of-concept fMRI study. Volume 2. Neuroimage: Reports; 2022. p. 100090. 2. 10.1016/j.ynirp.2022.100090 PMC9541093 36212800

[46] Benavides FD, et al. Cortical and subcortical effects of transcutaneous spinal cord stimulation in humans with tetraplegia. J Neurosci. 2020;40(13):2633–43. 31996455 10.1523/JNEUROSCI.2374-19.2020 PMC7096150

[47] Oh J, et al. Combinatorial effects of transcutaneous spinal stimulation and Task-Specific training to enhance hand motor output after paralysis. Top Spinal Cord Inj Rehabil. 2023;29(Suppl):15–22. 38174129 10.46292/sci23-00040S PMC10759855

[48] Brach JS, et al. Stance time and step width variability have unique contributing impairments in older persons. Gait Posture. 2008;27(3):431–9. 17632004 10.1016/j.gaitpost.2007.05.016 PMC2276116

[49] Patterson KK, et al. Evaluation of gait symmetry after stroke: a comparison of current methods and recommendations for standardization. Gait Posture. 2010;31(2):241–6. 19932621 10.1016/j.gaitpost.2009.10.014

[50] Goldie P, et al. Maximum voluntary weight-bearing by the affected and unaffected legs in standing following stroke. Clin Biomech Elsevier Ltd. 1996;11(6):333–42. 10.1016/0268-0033(96)00014-9 11415642

[51] Pai Y-C, et al. Alterations in weight-transfer capabilities in adults with hemiparesis. Phys Ther. 1994;74(7):647–57. 8016197 10.1093/ptj/74.7.647

[52] Dorsch S, Ada L, Canning CG. Lower limb strength is significantly impaired in all muscle groups in ambulatory people with chronic stroke: a cross-sectional study. Arch Phys Med Rehabil. 2016;97(4):522–7. 26615792 10.1016/j.apmr.2015.10.106

[53] Bastiaanse C, Duysens J, Dietz V. Modulation of cutaneous reflexes by load receptor input during human walking. Exp Brain Res. 2000;135:189–98. 11131503 10.1007/s002210000511

[54] Dietz V, Duysens J. Significance of load receptor input during locomotion: a review. Gait Posture. 2000;11(2):102–10. 10899663 10.1016/s0966-6362(99)00052-1

[55] Kozlovskaya I, et al. Role of support afferentation in control of the tonic muscle activity. Acta Astronaut. 2007;60(4–7):285–94.

[56] Gerasimenko Y, et al. Integration of sensory, spinal, and volitional descending inputs in regulation of human locomotion. J Neurophysiol. 2016;116(1):98–105. 27075538 10.1152/jn.00146.2016 PMC4961746

[57] Sayenko DG, et al. Self-assisted standing enabled by non-invasive spinal stimulation after spinal cord injury. J Neurotrauma. 2019;36(9):1435–50. 30362876 10.1089/neu.2018.5956 PMC6482915

[58] Angeli CA, et al. Altering spinal cord excitability enables voluntary movements after chronic complete paralysis in humans. Brain. 2014;137(Pt 5):1394–409. 24713270 10.1093/brain/awu038 PMC3999714

[59] Danner SM, et al. Human spinal locomotor control is based on flexibly organized burst generators. Brain. 2015;138(Pt 3):577–88. 25582580 10.1093/brain/awu372 PMC4408427

[60] Edgerton VR et al. Basic concepts underlying activity-dependent mechanisms in the rehabilitation of sensory-motor function after spinal cord injury. Spinal Cord Medicine: Third Edition, 2018.

[61] Hogan N. Adaptive control of mechanical impedance by coactivation of antagonist muscles. IEEE Trans Autom Control. 1984;29(8):681–90.

[62] Mawase F, et al. Motor learning enhances use-dependent plasticity. J Neurosci. 2017;37(10):2673–85. 28143961 10.1523/JNEUROSCI.3303-16.2017 PMC5354321

[63] Diedrichsen J, et al. Use-dependent and error-based learning of motor behaviors. J Neurosci. 2010;30(15):5159–66. 20392938 10.1523/JNEUROSCI.5406-09.2010 PMC6632748

[64] Krakauer JW, Mazzoni P. Human sensorimotor learning: adaptation, skill, and beyond. Curr Opin Neurobiol. 2011;21(4):636–44. 21764294 10.1016/j.conb.2011.06.012

[65] Classen J, et al. Rapid plasticity of human cortical movement representation induced by practice. J Neurophysiol. 1998;79(2):1117–23. 9463469 10.1152/jn.1998.79.2.1117

[66] Jax SA, Rosenbaum DA. Hand path priming in manual obstacle avoidance: evidence that the dorsal stream does not only control visually guided actions in real time. J Exp Psychol Hum Percept Perform. 2007;33(2):425–41. 17469977 10.1037/0096-1523.33.2.425

[67] Wong JD, et al. Can proprioceptive training improve motor learning? J Neurophysiol. 2012;108(12):3313–21. 22972960 10.1152/jn.00122.2012 PMC3544879

[68] Shadmehr R, Mussa-Ivaldi FA. Adaptive representation of dynamics during learning of a motor task. J Neurosci. 1994;14(5):3208–24. 8182467 10.1523/JNEUROSCI.14-05-03208.1994 PMC6577492

[69] Duysens J, Pearson K. The role of cutaneous afferents from the distal hindlimb in the regulation of the step cycle of thalamic cats. Exp Brain Res. 1976;24:245–55. 1253857 10.1007/BF00235013

[70] Duysens J, Pearson K. Inhibition of flexor burst generation by loading ankle extensor muscles in walking cats. Brain Res. 1980;187(2):321–32. 7370733 10.1016/0006-8993(80)90206-1

[71] Suggitt J, Symonds J, D’Amico JM. Safety and effectiveness of multisite transcutaneous spinal cord stimulation combined with Activity-Based therapy when delivered in a community rehabilitation setting: A Real-World pilot study. Neuromodulation: Technology at the Neural Interface; 2025. 10.1016/j.neurom.2025.01.005 39998450

[72] Rendos NK, et al. Verbal feedback enhances motor learning during post-stroke gait retraining. Top Stroke Rehabil. 2021;28(5):362–77. 32942960 10.1080/10749357.2020.1818480 PMC7969478

[73] Lee B-C, et al. The effect of vibrotactile Cuing on recovery strategies from a treadmill-induced trip. IEEE Trans Neural Syst Rehabil Eng. 2016;25(3):235–43. 10.1109/TNSRE.2016.2556690 28333619

